# *Histoplasma* Requires *SID1*, a Member of an Iron-Regulated Siderophore Gene Cluster, for Host Colonization

**DOI:** 10.1371/journal.ppat.1000044

**Published:** 2008-04-11

**Authors:** Lena H. Hwang, Jacob A. Mayfield, Jasper Rine, Anita Sil

**Affiliations:** 1 Department of Microbiology and Immunology, University of California, San Francisco, California, United States of America; 2 Department of Molecular and Cell Biology, California Institute of Quantitative Biosciences, University of California, Berkeley, California, United States of America; 3 Department of Molecular and Cell Biology, California Institute of Quantitative Biosciences, University of California, Berkeley, California, United States of America; University of Melbourne, Australia

## Abstract

The macrophage is the primary host cell for the fungal pathogen *Histoplasma capsulatum* during mammalian infections, yet little is known about fungal genes required for intracellular replication in the host. Since the ability to scavenge iron from the host is important for the virulence of most pathogens, we investigated the role of iron acquisition in *H. capsulatum* pathogenesis. *H. capsulatum* acquires iron through the action of ferric reductases and the production of siderophores, but the genes responsible for these activities and their role in virulence have not been determined. We identified a discrete set of co-regulated genes whose transcription is induced under low iron conditions. These genes all appeared to be involved in the synthesis, secretion, and utilization of siderophores. Surprisingly, the majority of these transcriptionally co-regulated genes were found clustered adjacent to each other in the genome of the three sequenced strains of *H. capsulatum,* suggesting that their proximity might foster coordinate gene regulation. Additionally, we identified a consensus sequence in the promoters of all of these genes that may contribute to iron-regulated gene expression. The gene set included L-ornithine monooxygenase (*SID1*), the enzyme that catalyzes the first committed step in siderophore production in other fungi. Disruption of *SID1* by allelic replacement resulted in poor growth under low iron conditions, as well as a loss of siderophore production. Strains deficient in *SID1* showed a significant growth defect in murine bone-marrow-derived macrophages and attenuation in the mouse model of infection. These data indicated that *H. capsulatum* utilizes siderophores in addition to other iron acquisition mechanisms for optimal growth during infection.

## Author Summary

Fungal infections are a growing public health threat, particularly for immunocompromised individuals such as people with AIDS, organ transplant recipients, and cancer patients. Present antifungal therapies are often highly toxic and resistance to these therapies continues to rise. *Histoplasma capsulatum* is a pathogenic fungus that infects humans, causing pulmonary and systemic disease. It is the most common cause of fungal respiratory infection in the world, and is endemic to the Mississippi and Ohio River valleys of the United States. *H. capsulatum* produces small molecules, called siderophores, to acquire iron, an essential nutrient. We have identified genes that are involved in the synthesis of siderophores in this fungus and have found that siderophore production in *H. capsulatum* is important for its virulence. Since siderophore production is confined to microbes and plays no role in human biology, it is an excellent target for rational drug design.

## Introduction

Iron acquisition is critical to cellular function and survival. During infection of mammals, the host limits the access of iron to microbial pathogens by a variety of means [1]. In turn, pathogens utilize a number of strategies to acquire iron in the face of iron restriction by the host. Here we investigate the role of siderophore-mediated iron acquisition in the fungal pathogen Histoplasma capsulatum, which parasitizes host macrophages during infection.

*Histoplasma capsulatum* is a dimorphic, fungal pathogen that causes respiratory and systemic disease in humans. Infection of mammals is initiated by inhalation of fungal spores from soil in regions of the U.S. where the organism is endemic. Once in the host, H. capsulatum grows in a budding yeast form that colonizes alveolar macrophages. Yeast cells replicate within the macrophage phagolysosome, but the molecular mechanisms governing survival within host cells remain largely undefined [2].

Replication of *H. capsulatum* within macrophages is dependent on the availability of iron. During infection of murine peritoneal and human monocyte-derived macrophages, addition of the iron chelator deferoxamine inhibits the intracellular growth of *H. capsulatum*. This inhibition is suppressed by the addition of iron-rich transferrin (holotransferrin), indicating that active iron acquisition is a critical determinant of intracellular growth [3],[4]. In addition, the adaptive immune response to *H. capsulatum* infection, which triggers production of the cytokine interferon-gamma (IFNγ) by T-cells [5], may curtail intracellular fungal growth by limiting iron acquisition by the fungus. Treatment of murine peritoneal macrophages with interferon-gamma (IFNg) causes growth restriction of *H. capsulatum* which, in turn, can be reversed by addition of holotransferrin [3]. IFNγ downregulates surface transferrin receptors, suggesting that a major means by which IFNγ inhibits intracellular fungal growth is via iron limitation.

These studies indicate that iron acquisition plays a critical role in *Histoplasma* virulence. However, although several biochemical activities have been identified in *H. capsulatum*, little is known about the genes that regulate iron accumulation. *H. capsulatum* is known to secrete hydroxamate siderophores that act as low-molecular-weight ferric iron chelators under low iron conditions [6],[7]. Additionally, iron limitation also induces reductive iron assimilation, including ferric reductase activity [8].

To assess the role of these iron acquisition mechanisms in virulence, we identified *H. capsulatum* genes that function in siderophore-mediated iron acquisition. These genes were transcriptionally induced under low iron conditions and contained a common putative regulatory site in their upstream regions that might govern their coordinate expression. Inspection of the sequences revealed that these genes were clustered together in the genome, and thus defined a secondary metabolite gene cluster involved in siderophore biosynthesis. Disruption of one of the genes of this pathway, *SID1*, resulted in elimination of hydroxamate siderophore synthesis, diminished growth of *H. capsulatum* within macrophages, and compromised virulence in mice. These data indicated that iron scavenging though siderophore production facilitates the parasitic growth of *H. capsulatum.*

## Results

### Transcriptional response to low iron

**Figure 1:**
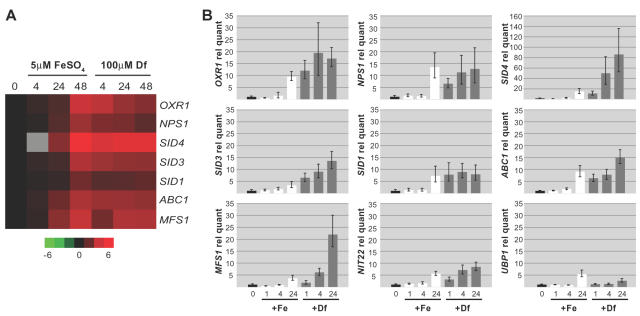
Iron Regulated Gene Expression of Siderophore Biosynthesis and Utilization Genes. A. Iron-dependent gene expression cluster from whole genome oligonucleotide microarray analysis of G217B yeast grown under iron replete (5 µM FeSO_4_) and iron limited (100 µM deferoxamine mesylate) conditions for 0, 4, 24, and 48 hours, as described in Materials and Methods. Red indicates up-regulated genes, green indicates down-regulated genes, black indicates genes that do not change in expression, and gray indicates that no data are available. Microarray time courses were repeated with three biological replicates. A representative experiment is shown. Similar results were seen for iron replete conditions of either 5 or 10 µM FeSO_4_. B. Quantitative RT-PCR of siderophore biosynthesis and utilization genes. Total RNA from the 0, 1, 4, and 24 hr time points used in the microarray analysis were analyzed for relative transcript levels using QRT-PCR. Transcript levels were normalized to *ACT1* transcript. Relative quantities were normalized to t = 0 values (black bars). For the 1, 4, and 24 hr time points, white bars indicate growth in medium supplemented with FeSO_4_ (+Fe), and gray bars indicate growth in deferoxamine (+Df).

The genes involved in iron acquisition are tightly regulated at the level of transcription in most microorganisms. To identify genes that are transcriptionally regulated by iron limitation conditions in *H. capsulatum*, we grew *H. capsulatum* (strain G217B) yeast cells under iron-limiting (100 µM deferoxamine mesylate) or iron-replete (5 or 10 µM FeSO_4_) conditions for various periods of time. The transcriptional profiles of these samples were compared using whole-genome oligonucleotide microarrays. We found seven genes that were transcriptionally induced under conditions of iron limitation and named them according to the putative functions of the corresponding proteins **(** **Figure 1A**). These genes included L-ornithine monooxygenase (*SID1*, previously named *LOM1* [9], EU253976), the enzyme catalyzing the first committed step in extracellular and intracellular siderophore production in other organisms; an acetylase (*SID3,* EU253977); an acid co-A ligase (*SID4,* EU253978); a non-ribosomal peptide synthase (*NPS1,* EU253973); an oxidoreductase (*OXR1,* EU253974); a major facilitator superfamily (MFS) transporter (*MFS1,* EU253970) [10]; and an ATP-binding cassette transporter (*ABC1*, EU253969) [10].

Based on precedent from other fungi, these genes were likely to have roles in the production, transport, and utilization of siderophores, and could comprise the entire pathway of hydroxamate siderophore production [11]–[13]. *SID3* is orthologous to SidF in *Aspergillus fumigatus*, an N^5^-transacylase involved in the synthesis of fusarinine and triacetylfusarinine [13]. *NPS1* is orthologous to the non-ribosomal peptide synthases involved in extracellular siderophore production in ascomycetes [13],[14]. *MFS1* is homologous to *mirB* of *Aspergillus nidulans*, a siderophore transporter [15]. And finally, *ABC1* contains homology to MTABC3, a mammalian ABC transporter involved in iron homeostatis [16].

Quantitative RT-PCR (QRT-PCR) analysis confirmed that all seven genes were transcriptionally induced during iron deprivation (**Figure 1B**). Both the microarrays and QRT-PCR analysis also showed that transcription of these genes began to be induced even in the presence of iron supplementation at late time points, suggesting that iron eventually becomes limiting under these conditions, or perhaps that the induction of genes involved in siderophore synthesis is highly sensitive to a subtle decrease in iron availability.

**Figure 2:**
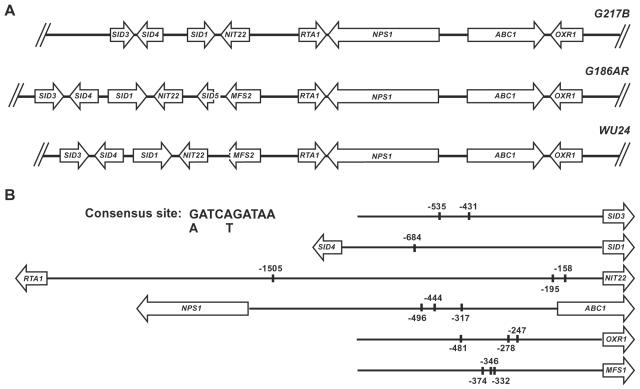
Genome Organization and Consensus Site Identification for Siderophore Biosynthetic Genes. A. Schematic showing genome structure of siderophore biosynthesis and utilization genes in *H. capsulatum* strains G217B, G186AR, and WU24. B. Location and sequence of putative consensus site in promoters of siderophore genes in the G217B strain. One kilobase of sequence 5′ to the open reading frame for each gene is represented by a black line, except for the divergent genes: *SID4* and *SID1*, with an intergenic region of 1036 bases; *RTA1* and *NIT22,* with an intergenic region of 2762 bases; and *NPS1* and *ABC1*, with an intergenic region of 1677 bases. The location of the consensus sequence is marked with a short vertical line and the associated number represents the number of base pairs from the start codon of the downstream gene. The 5′ to 3′ orientation of the consensus site is not indicated.

Surprisingly, all but one of these iron-regulated genes were located adjacent to each other in an approximately 25 kb region of the genome of the G217B strain of *H. capsulatum* (**Figure 2A**). Only *MFS1* is encoded by a distinct genomic region. This genomic cluster of iron-regulated genes was also present in the two other sequenced strains of *H. capsulatum:* G186AR (Genome Sequencing Center, Washington University, St. Louis, MO) and WU24 (Broad Institute, Massachusetts Institute of Technology, Cambridge, MA). In G186AR, an additional MFS transporter, *MFS2* (EU253971), as well as a second acetylase (*SID5,* EU253979), was found within the putative iron-regulated gene cluster. WU24 contained the 5′ end of the *MFS2* gene, but did not contain *SID5*. We observed a similar genome structure in the closely related fungus *Coccidioides immitis* (data not shown), but only a subset of the genes were found adjacent to each other in other fungal genomes (see discussion).

Two of the genes located within this genomic cluster, *NIT22* (encoding a dehydratase, [9], EU253972) and *RTA1* (encoding a predicted protein containing an RTA domain, resistance to 7-aminocholesterol, EU253975), did not appear to be iron regulated by microarray. We used QRT-PCR analysis to determine whether these genes were regulated differentially in response to iron levels. However, whereas *RTA1* expression was not detected by QRT-PCR under either iron-rich or iron-poor conditions (data not shown), *NIT22* expression was induced under iron limitation (**Figure 1B**). Unlike the other iron-regulated genes in this genomic cluster, *NIT22* did not encode a protein with homology to known siderophore-biosynthesis genes. Thus, its induction under conditions of iron limitation may reflect a previously unidentified role of its dehydratase activity in siderophore biosynthesis. To determine if the iron-dependent regulation of this gene cluster extended beyond the genes we identified by microarray, we also examined the expression of *UBP1* (encoding a ubiquitin C-terminal hydrolase), a gene approximately 6kb upstream of *OXR1. UBP1* expression was not regulated by iron levels (**Figure 1B**). In addition, we used QRT-PCR to determine that genes found in the iron cluster of G186AR were also transcriptionally regulated by iron levels (**Figure S1**).

### Identification of a putative regulatory site

Since the genes in this siderophore biosynthetic cluster were transcriptionally co-regulated in response to iron levels, we used Multiple Em for Motif Elicitation (MEME) to identify conserved sequences in the upstream regions of these genes from both the G217B and G186AR strains of *H. capsulatum*. A consensus site, 5′-(G/A)ATC(T/A)GATAA-3′, was present at least once in the 5′ regions of all of the genes in the siderophore biosynthetic cluster (**Figure 2B**). Interestingly, this consensus contained an HGATAR sequence, the recognition site for fungal GATA transcriptional regulators [17], some of which are known to regulate the expression of siderophore-biosynthesis genes in other fungi [18]–[20]. In *H. capsulatum*, an ortholog of these GATA factors, Sre1, was shown to bind *in vitro* to the consensus site we identified here, suggesting that Sre1 limits expression of the siderophore biosynthetic gene cluster under iron-replete conditions (Chao et al, submitted).

### Disruption of *SID1* eliminated siderophore production

**Figure 3:**
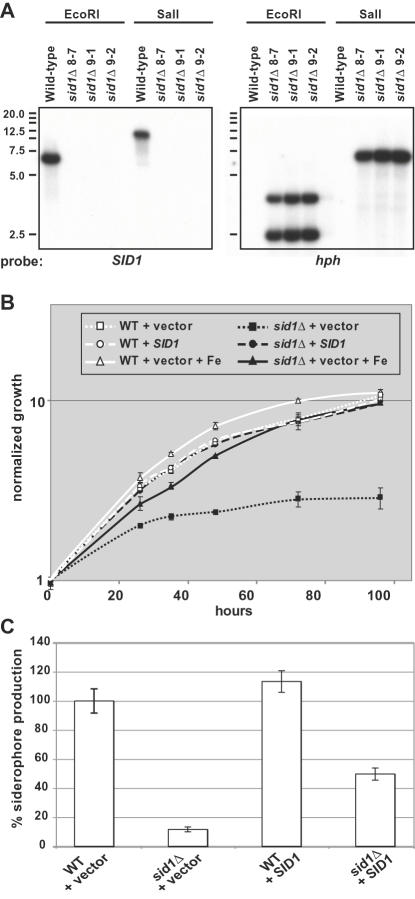
Disruption of *SID1* Resulted in Iron-Dependent Growth and Defects in Siderophore Production. A. Southern analysis of *SID1* disruption. Genomic DNA from parental strain (WU8-Wild-type) and potential *sid1Δ::hph* strains (HcLH25, HcLH26, and HcLH27) was digested by EcoR*I* or Sal*I*, separated by gel electrophoresis, blotted and hybridized to probes containing either the open reading frame of *SID1* or the *hph* gene. For the *SID1* probe, fragments of the expected sizes of 6.4 kb (EcoR*I*) and 12.2 kb (Sal*I*) are seen in the DNA from the parental strain. For the *hph* probe, fragments of the expected sizes of 3.6 kb and 2.3 kb (EcoR*I*) and 6.8 kb (Sal*I*) are seen in the DNA from *sid1Δ* strains. B. Growth curves of HcLH95 (WT+vector), HcLH97 (*sid1Δ*+vector), HcLH103 (WT+SID1), and HcLH106 *(sid1Δ+SID1*). Yeast cells were grown in mRPMI medium (pH 6.5) without or with 5 µM FeSO_4_ added (+Fe). Growth and siderophore production experiments were repeated at least three times with two independent *sid1Δ* strains and four independent complementation strains. Samples for OD_600_ were taken in triplicate. The mean+/−standard deviation is indicated. A representative experiment is presented. C. Siderophore production of strains. Yeast cells (same strains used in B) were grown in mRPMI medium (pH 6.5) for 24 hours and culture supernatant was examined for presence of siderophores using chrome azurol S assay as described in Materials and Methods. Samples were taken in triplicate. The mean+/−standard deviation is indicated. Results are given as percentage of siderophore production compared with WT+vector.

To test whether this pathway was important for siderophore production, we disrupted *SID1*, which encodes the enzyme that catalyzes the first committed step in siderophore production. The gene disruption was performed in the wild-type G186AR*ura5Δ* strain background and confirmed by PCR (data not shown) and Southern blot analysis (**Figure 3A**). The *sid1Δ* strain grew with normal kinetics under iron-replete conditions, but displayed a growth defect under conditions of iron limitation (**Figure 3B**). Consistent with its poor growth under iron-limiting conditions, the *sid1Δ* strain also failed to produce siderophores (**Figure 3C**).

To insure that the observed growth defect was due to disruption of *SID1*, we complemented the *sid1Δ* strain with a wild-type copy of the *SID1* gene. *SID1*, including all intergenic regions 5′ and 3′ of the open reading frame, was cloned into an integrating *Agrobacterium tumefaciens* T-DNA vector (pED2). This construct was randomly integrated into the wild-type genome and into two independent *sid1Δ* isolates. The *SID1* gene fully complemented the growth defect in low-iron medium (**Figure 3B**), but only partially complemented siderophore production (**Figure 3C**) for unknown reasons. QRT-PCR analysis revealed that *SID1* transcript accumulation was similar under iron-limiting conditions in the wild-type and complemented strains (data not shown). We also introduced a *SID1* complementation construct on a high-copy episomal plasmid, but these transformants expressed *SID1* at very low levels and showed little complementation of siderophore production (data not shown), suggesting that integration into the genome may be required for normal levels of *SID1* transcription.

### *SID1* is important for virulence

**Figure 4:**
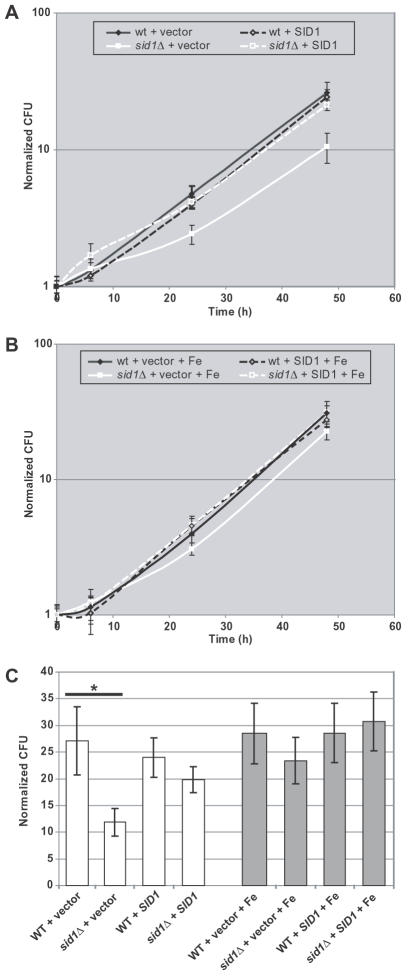
Disruption of *SID1* Results in Impaired Growth During Infection of Macrophages. HcLH95 (WT+vector), HcLH97 (*sid1Δ*+vector), HcLH103 (WT+*SID1*), and HcLH106 (*sid1Δ+SID1*) were used to infect C57BL/6J bone-marrow-derived macrophages at a multiplicity of infection of 5. Macrophages were lysed at t = 0, 6, 24, and 48 hours, and plated for *H. capsulatum* colony-forming units (CFUs) on HMM. CFUs were normalized to the zero time point. Infected macrophages were incubated in either BMM (A) or BMM+100 µM FeSO_4_ (B) Data from a representative time course is shown. The average of three platings+/−standard error of the mean is shown. Significance was determined using ANOVA with Bonferroni Multiple Comparisons Test. Comparison of wt+vector and *sid1Δ*+vector at 48 hours has a p<0.001. The experiment was repeated three times with two independently isolated *sid1Δ* strains, as well as four independently isolated *sid1Δ+SID1* strains. (C) The average CFU at 48 hours of three independent infections+/−standard error of the mean is shown. White bars indicate CFUs from infected macrophages incubated in BMM, grey bars indicate macrophages incubated in BMM+100 µM FeSO_4_. Significance was determined for the influence of genotype (WT versus *sid1Δ*) and iron using three-way ANOVA. Genotype influence, *: p-value<0.05.

In other pathogens, iron acquisition from the host is a critical virulence determinant. To examine the role of siderophore-mediated iron acquisition in *H. capsulatum* infection, we infected bone-marrow-derived murine macrophages (BMDMs) with wild-type, *sid1Δ*, and complemented strains. At zero, 6, 24, and 48 hours following infection, the macrophages were lysed and colony-forming units (CFUs) of *H. capsulatum* cells were determined (**Figure 4A**). Wild-type *H. capsulatum* cells were able to proliferate within the macrophages as expected. Although the *sid1Δ* strains were also able to grow intracellularly, they reached only ∼40% of wild-type levels by 48 hours after infection (p<0.001). The doubling time of wild-type cells in macrophages was 10 hours while the *sid1Δ* strain doubled in 14 hours. Addition of 100 µM FeSO_4_ to infected macrophages reversed this phenotype, strongly suggesting that the proliferation defect in macrophages was due to the decreased ability of the mutant strains to acquire iron (**Figure 4B**). The complemented strains did not display a growth defect in macrophages, indicating that they made sufficient siderophores to permit wild-type growth during macrophage infection (**Figure 4A**). The growth defect of the mutant strain and complementation of the defect with either reconstitution of the wild-type gene or the addition of iron was consistent over several infections (**Figure 4C**).

**Figure 5:**
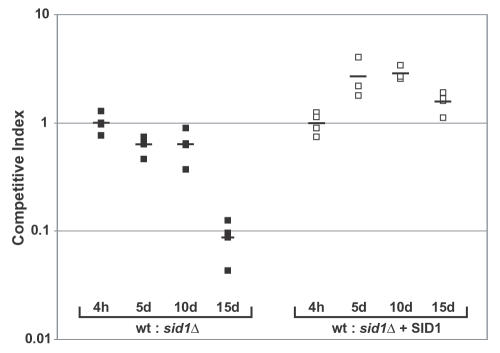
Disruption of *SID1* Causes a Growth Defect in Mice. C57BL/6J mice were infected intranasally with an effective inoculum of 1×10^4^ CFU of an equal mix of hcLH95 and hcLH97 (wt:*sid1Δ,* black boxes) or hcLH95 and hcLH106 (wt: *sid1Δ+SID1*, white boxes). Boxes indicate the competitive index (CI) for individual mice as determined in Materials and Methods. Horizontal bars indicate the means of CI values. A pairwise comparison of the 15-day CI for the wt:*sid1Δ* and wt: *sid1Δ+SID1* infections by the Mann-Whitney/Wilcoxon test revealed that they were significantly different (p = 0.0286). Significance for WT:*sid1Δ* time course was determined using Kruskal-Wallis test (ANOVA) with Dunn's Multiple Comparisons test. This analysis showed that the CI at t = 4 hr and t = 15 days was significantly different (p<0.01).

To investigate whether siderophore production plays a role in animal infections, we first infected C57BL/6J mice intranasally with a lethal dose (effective inoculum of ∼1×10^5^ CFU) of either wild-type, *sid1Δ,* or complemented strains. No significant differences in pulmonary fungal burden were observed under these conditions (data not shown). However, since the literature suggested that assessing the virulence of a mutant strain in competition with wild-type can provide a more sensitive assay for pathogenesis defects [21],[22], we decided to perform a competitive infection with wild-type, *sid1Δ,* and the complemented strains. We infected C57BL/6J mice intranasally with a sublethal dose (effective inoculum of ∼1×10^4^ CFU) of an equal mix of either wild-type and *sid1Δ* or wild-type and *sid1Δ*+*SID1* strains. Lung homogenates from multiple time points were analyzed for CFUs and ratios of wild-type, *sid1Δ*, and complemented strains were calculated. The competitive index, which reflects the defect of a particular strain with respect to the wild-type strain, was determined as described in Materials and Methods. We observed that the *sid1Δ* strain showed a significant defect in pulmonary colonization compared to wild-type cells (**Figure 5**). This defect began at day 5, with a severe defect apparent at day 15 (p<0.01). This phenotype was reversed in the complemented strain, which accumulated to slightly higher levels than the wild-type strain for unknown reasons (p<0.05). These data indicated that siderophore production is important for optimal growth of *H. capsulatum* in mouse lungs.

## Discussion

Iron acquisition is essential for the growth of most microorganisms and occurs primarily by two mechanisms: siderophore production and reductive iron assimilation [23]. Both of these processes are induced by *H. capsulatum* during iron limitation [6],[8], but their role in cell growth and virulence has not been previously investigated. In this study, we determined the role of siderophore production in growth under conditions of iron limitation as well as during infection. We identified a siderophore biosynthetic gene cluster that was transcriptionally induced in response to iron limitation. Disruption of siderophore production resulted in iron-dependent growth in culture and during macrophage infection, and caused a growth defect in mice.

Presumably the *sid1Δ* mutant shows a strong pulmonary colonization defect in competition with wild-type because it has a reduced capacity for iron acquisition *in vivo*. The *in vivo* growth defect caused by lack of siderophore production is most pronounced at 15 days post infection. Interestingly, the peak of IFN-γ production by T cells in response to *H. capsulatum* infection occurs at day 14 [24]. Since one of the functions of IFN-γ is to restrict iron, perhaps siderophore production is primarily required for iron acquisition during the latter stages of infection, whereas redundant iron acquisition mechanisms, such as reductive iron assimilation, might play a role early in infection.

In other fungal pathogens, siderophore production and reductive iron assimilation, which is usually mediated by ferric reductase activity, play differential roles in pathogenesis. For example, siderophore production is essential for virulence in *Aspergillus fumigatus*, but reductive iron assimilation is neither necessary nor sufficient for normal growth and survival in the host [25]. Similarly, in the phytopathogens *Cochliobolus miyabeanus*, *Alternaria brassicicola, Cochliobolus heterostrophus*, and *Fusarium graminearum*, extracellular siderophore production is required for full virulence [14]. In contrast, deletion of the *SID1* gene in *U. maydis* does not affect virulence in maize [26]; instead, reductive iron assimilation is required for virulence [27]. This also true in *Candida albicans*, where ferric reductase activity is required for systemic infection [25],[28].

Ferric reductase activity has been clearly demonstrated in *H. capsulatum* [8], though the relevant genes that encode these activities have not been identified, and thus their role in virulence has not been assessed. Perusal of the genome revealed seven putative ferric reductase genes, though none of these candidates were observed to be upregulated by iron limitation in our experiments. Nonetheless, any or all of these genes could contribute to pathogenesis in the host.

In addition to identifying a role for siderophore production in virulence, this study revealed several interesting regulatory properties of siderophore-biosynthesis genes. First, inspection of the sequences upstream of each gene revealed a consensus sequence that was present at least once per gene. The expression of several fungal orthologs of *SID1* is repressed by GATA-type negative regulators that recognize the HGATR motif [19],[20],[29]. Interestingly, the consensus sequence we identified, 5′-(G/A)ATC(T/A)GATAA-3′, contained an HGATAR motif, and was shown to bind an *H. capsulatum* GATA factor *in vitro* (Chao et al., submitted). We are now investigating whether these regulatory sequences are necessary to confer gene regulation in response to iron levels *in vivo*.

Second, the siderophore biosynthesis genes were located adjacent to each other in the genome, and thus comprise the first secondary metabolite gene cluster defined in *H. capsulatum*. We observed similar clustering in the genome of the closely related systemic dimorphic fungal pathogen *C. immitis*, but it is not evident to nearly the same extent for siderophore biosynthesis genes in other sequenced fungal genomes [27],[30],[31].

Third, we could not express significant levels of *SID1* from an episomal plasmid, suggesting that integration of the gene may be critical for normal expression levels. Additionally, integration of *SID1* at random sites in complementation strains resulted in partial complementation of siderophore production, suggesting that optimal function might be achieved only with expression from the original genomic locus. Unfortunately, targeted integration is extremely challenging in *H. capsulatum*, making it difficult to directly test this hypothesis.

The potential significance of the *H. capsulatum* gene cluster with regards to gene regulation is intriguing. One possibility is that clustering in the genome facilitates local changes in chromatin structure that allow a regional change in promoter accessibility. This type of regulation is reminiscent of transcriptional control of secondary metabolite clusters in *Aspergillus* species, where transcriptional accessibility of genes in the cluster is controlled by the putative methyltransferase LaeA [32],[33]. Perhaps local control of chromatin structure allows a rapid and coordinated transcriptional switch in response to changes in iron levels.

## Materials and Methods

### Strains and culture conditions

*Histoplasma capsulatum* strains G217B (ATCC 26032) and G186AR*ura5Δ* (WU8), all kind gifts from the laboratory of William Goldman, Washington University, St. Louis, as well as strains generated in this study (Table 1) were grown in HMM broth or plates [34], or in mRPMI broth [RPMI 1640 medium without phenol red or bicarbonate (Invitrogen, www.invitrogen.com), supplemented with 1.8% dextrose (Fisher Scientific, www.fishersci.com), 25 mM HEPES pH 6.5 or pH 7.5 (Fisher Scientific), and 100 units/mL of both penicillin and streptomycin (UCSF Cell Culture Facility, www.ccf.ucsf.edu), modified from [8]]. Unlike HMM, mRPMI contained no added iron, but did not eliminate trace amounts of iron, though cultures grown in mRPMI medium were incubated in plastic flasks to reduce trace iron contamination. Media was supplemented with various concentrations of FeSO_4_ (Fisher Scientific) as described in the text. HMM and mRPMI media were supplemented when needed with 200 µg/mL hygromycin (Roche, www.roche.com) and 200 µg/mL uracil (Sigma-Aldrich, www.sigmaaldrich.com). Cultures were grown at 37°C under 5% CO_2_.

**Table 1:** Strain List.

Strains	Genotype
ATCC 26032	G217B wt
WU8	G186AR *ura5Δ*
HcLH25	G186AR *ura5Δ sid1Δ(8−7)::hph*
HcLH26	G186AR *ura5Δ sid1Δ(9−1)::hph*
HcLH27	G186AR *ura5Δ sid1Δ(9−2)::hph*
HcLH95	G186AR *ura5Δ zzz::URA5*
HcLH97	G186AR *ura5Δ sid1Δ::hph zzz::URA5*
HcLH103	G186AR *ura5Δ zzz::SID1-URA5*
HcLH106	G186AR *ura5Δ sid1Δ::hph zzz::URA5*

*zzz* indicates integration into unknown location in genome.

For microarray studies, an initial culture of G217B yeast cells was grown in 5 mL HMM, and then passaged 1∶25 into 80 mL HMM. After 3 days of growth, the culture was pelleted, washed in 80 mL of PBS, and resuspended in 1 L of mRPMI pH 7.5 supplemented with 5 µM FeSO_4_. After 24 hours of growth, 200 mL of culture was harvested for the zero time point. Then the culture was split into 2×400 mL, and an additional 5 µM or 10 µM FeSO_4_ was added to one culture and 100 µM deferoxamine mesylate (Sigma-Aldrich) was added to the other. At each time point, 100 mL of culture was harvested and processed as below.

For growth, siderophore production, and QRT-PCR assays in G186AR based strains, cells were grown in 5 mL HMM. When the cultures reached late log phase, they were sonicated twice for 3 seconds to disperse cells and then passaged 1∶25 into HMM. After 24 hours of growth, they were pelleted, washed in an equal volume of PBS, and resuspended in an equal volume of mRPMI pH 6.5. For growth curves, triplicate samples of 1 mL of cells were taken at each time point, sonicated twice for 3 seconds, and dilutions were measured by spectrophotometer at OD_600_. For QRT-PCR, after resuspension in mRPMI pH 6.5, cells were grown for an additional 24 hours. At that point, a t = 0 sample was harvested, then the culture was split. One culture was maintained in mRPMI pH 6.5 media with no additions, the other was treated with 5 µM FeSO_4_. Cells were harvested at 1, 4, and 24 hours and total RNA was isolated using a guanidine thiocyanate lysis protocol as previously described [10].

### Electrotransformation of DNA

Approximately 100 ng of PacI-linearized plasmid DNA with exposed telomere ends was transformed into yeast cells as previously described [35].

### Agrobacterium Transformation

*H. capsulatum* yeast cells were transformed using *Agrobacterium*-mediated gene transfer as described previously [36]. Briefly, the *A. tumefaciens* strain (LBA1100, a kind gift of Thomas Sullivan and Bruce Klein with permission from Paul Hooykas (Leiden University, Leiden, The Netherlands)) transformed with the desired plasmid was induced overnight with 200 µM acetosyringone (AS, from Sigma-Aldrich). *H. capsulatum* yeast cells were harvested from 4 day patches on HMM+uracil plates and diluted to 5×10^8^ cells/mL. Equal volumes of the *H. capsulatum* and *A. tumefaciens* cultures were mixed, and 400 µL of the mix was spread onto BiodyneA nylon membranes (Pall Gelman, www.pall.com) on IM agarose plates containing 200 µM AS and 200 µg/mL uracil. Co-cultivation plates were incubated at 28°C for 3 days. The membranes were then transferred onto HMM plates with no added uracil and incubated at 37°C for 2 to 3 weeks. Strains WU8, HcLH25, and HcLH26 were transformed with pVN61 (vector control marked with *URA5*) and pED2 (*SID1* complementation marked with *URA5*). All of the transformed strains show similar *in vitro* growth in liquid and solid media without uracil.

### Microarrays

Cultures of *H. capsulatum* were harvested by filtration, and total RNA was isolated using a guanidine thiocyanate lysis protocol as previously described [10]. This RNA was used for both microarrays as well as QRT-PCR. For microarrays, polyadenylated RNA was purified from total RNA using an Oligotex mRNA kit (Qiagen Inc.,www.qiagen.com). cDNA synthesis from polyA-selected RNA and fluorescent labeling was performed as described previously [37]. Briefly, an equal mass of RNA from each time point was pooled to generate a reference sample and labeled with Cy3. cDNA from each individual time point was labeled with Cy5 and competitively hybridized versus the reference pool using *H. capsulatum* G217B 70-mer oligonucleotide microarrays. The microarrays contain a single 70-mer oligo for each predicted gene in the G217B genome, as well as two 70-mer oligos for low confidence genes.

Arrays were scanned on a GenePix 4000B scanner (Axon Instruments/Molecular Devices, www.moleculardevices.com) and analyzed using GENEPIX PRO, version 6.0 (Molecular Devices), Spotreader (Niles Scientific, www.nilesscientific.com), NOMAD 2.0 (http://nomad2.ucsf.edu/NOMAD/nomad-cgi/login.pl), CLUSTER [38], and Java Treeview 1.0.12 (available at http://sourceforge.net/project/showfiles.phpgroup_id84593). To eliminate elements with low signal, we did not analyze elements for which the sum of the medians for the 635-nm and 532-nm channels was ≤500 intensity units. All of the time points were normalized relative to the zero time point.

### Quantitative RT-PCR

Total RNA was treated with DNaseI (Promega, www.promega.com). cDNA was synthesized from 3.3 µg of DNaseI-treated RNA using Stratascript reverse transcriptase (Stratagene, www.stratagene.com) and oligo-dT. Quantitative PCR was performed on 1∶100 dilutions of cDNA, except for *MFS2* and *SID5* reactions, which used 1∶40 dilutions of cDNA. The reactions included 1.5 mM MgCl_2_, 1× Amplitaq buffer, 0.6 units Amplitaq Gold (Applied Biosystems, www.appliedbiosystems.com), 1× SYBR Green (Molecular Probes, probes.invitrogen.com), and 200 nM primers. Reactions were performed on the Mx3000P QPCR system (Stratagene, www.stratagene.com) with Comparative Quantitation (with dissociation curve) program, using actin (*ACT1*) as the normalizing transcript. Cycling parameters were 95°C for 10 minutes, then 40 cycles of 95°C (30 s), 57°C (1 min), 72°C (30 s) followed by dissociation curve analysis. All reactions were performed in triplicate. A calibrator sample made from an equal mix of each RNA sample was included on each plate. Reactions were analyzed using MxPro software. Primer sequences are included in supplemental material (Table S1).

### Accession Numbers

The *H. capsulatum* GenBank/NCBI (http://www.ncbi.nlm.nih.gov/) nucleotide sequences for the genetic loci described in this publication are *ABC1* (EU253969), *MFS1* (EU253970), *MFS2* (EU253971), *NIT22* (EU253972), *NPS1* (EU253973), *OXR1* (EU253974), *RTA1* (EU253975), *SID1* (EU253976), *SID3* (EU253977), *SID4* (EU253978), and *SID5* (EU253979).

### Consensus site identification

Promoter regions (including 1 kb upstream of ATG, or entire intergenic regions for *SID4/SID1* and *NPS1/ABC1)* of *SID3, SID4, SID1, NIT22, NPS1, ABC1, OXR1,* and *MFS1* from G217B and G186AR were submitted for MEME (Multiple Em for Motif Elicitation) analysis at http://meme.sdsc.edu. The length range for consensus site was set between 6 and 50 base pairs.

### Disruption of *SID1*

A positive-negative selection strategy was utilized to disrupt *SID1* in G186AR*ura5Δ* (WU15), as described in [39]. Originally, we attempted to disrupt this gene in the G217B*ura5-23* strain, but we were unsuccessful. A disruption construct, pLH36, was made containing 1213 bps 5′ and 943 bps 3′ of the *SID1* open reading from G217B. The open reading frame was replaced with the hygromycin resistance gene, *hph* under the control of the *Aspergillus nidulans gpd* promoter. This construct was introduced into G186AR*ura5Δ* (WU15) by electrotransformation. Seven independent transformants were colony purified, inoculated into HMM+hygromycin+uracil+20 µM FeSO_4_ medium and passaged 3 times (1∶25 dilution) once the cultures had reached mid log phase. After the final passage, serial dilutions of the cells were plated onto HMM (pH 4.5)+1 g/L 5-Fluoroorotic acid (5-FOA, Zymo Research, www.zymoresearch.com) +20 µM FeSO_4_ agarose plates. Genomic DNA from colonies on 5-FOA plates was tested by PCR for the disruption. Potential gene disruptions were further tested by Southern analysis.

### Quantification of siderophores

Secretion of siderophores was detected using chrome azurol S (CAS) [40]. Briefly, 0.5 mL of culture supernatant was mixed with 0.5 mL of CAS assay solution (600 µM hexadecyltrimethyl ammonium (Sigma Aldrich), 15 µM FeCl_3_ (Sigma Aldrich), 150 µM CAS (Sigma Aldrich), 500 mM anhydrous piperazine (Fluka), 750 mM HCl (Fisher Scientific), and 4 mM 5-sulfosalicylic acid (Sigma Aldrich)), incubated for 1 hour, and the OD_630_ was measured with a spectrophotometer.

### Macrophage culture and infections

Bone-marrow-derived macrophages (BMDM) were isolated from femurs of 6- to 8-week old female C57BL/6J mice. The bone marrow was eluted using BMM (Bone Marrow Macrophage) medium, which consists of Dulbecco's Modified Eagle Medium, D-MEM High Glucose (UCSF Cell Culture Facility), 20% Fetal Bovine Serum (Hyclone, Thermo Fisher, www.hyclone.com), 10% v/v CMG supernatant (the source of CSF-1), 2 mM glutamine (UCSF Cell Culture Facility), 110 µg/mL sodium pyruvate (UCSF Cell Culture Facility), penicillin and streptomycin (UCSF Cell Culture Facility). The bone marrow cells were cultured for 6 days at 37°C with 5% CO_2_. The cells were harvested with cold PBS (without Mg and Ca), and frozen in BMM+10% DMSO.

For infections, *H. capsulatum* cells were grown to late log phase in HMM. The cells were pelleted and resuspended in BMM medium, sonicated twice for 3 seconds, and counted by hemacytometer. Approximately 1×10^6^ *H. capsulatum* cells were used to infect 2×10^5^ BMDM in 24-well cell culture dishes. After a 1 hr incubation, the infected macrophages were washed twice with D-MEM High Glucose and then incubated in 500 µL fresh BMM medium with or without 100 µM FeSO_4_ at the zero time point. At 6, 24 and 48 hrs, the medium was removed from the wells and 500 µl H_2_O was added. After 5 min incubation, the lysed macrophages were transferred to a 1.5 mL eppendorf and sonicated for 3 sec. Dilutions were plated onto HMM plates to determine CFUs. CFUs were normalized to the zero time point. Significance was determined using ANOVA with Bonferroni Multiple Comparisons Test.

### Competitive index mouse infections

*H. capsulatum* strains hcLH95 (wild-type), hcLH97 (*sid1Δ::hph*), and hcLH106 (*sid1Δ::hph+SID1)* were grown in 5 mL of HMM. Cells were passaged once and grown until late log phase. 1 mL of culture was sonicated twice for 3 seconds to fully disperse cells then washed twice with cold PBS. Cell concentration was determined by hemacytometer. Mice were infected intranasally with 5×10^4^ cells in 25 µL PBS containing an equal proportion of hcLH95 and hcLH97 (wild-type and *sid1Δ::hph*) or hcLH95 and hcLH106 (wild-type and *sid1Δ::hph*+*SID1*) followed by 5 µL of PBS chase. Four mice were used for each time point.

Lungs were homogenized in 5 mL of HMM. Serial dilutions were plated onto both HMM and HMM+hygromycin plates in order to distinguish *sid1Δ::hph* and *sid1Δ::hph*+*SID1* strains from the wild-type strain. Enumeration of wild-type, *sid1Δ*, and *sid1Δ*+*SID1* yeast allowed for the determination of competitive index ratio (CI) using the following formula: CI = (mutant output/wild-type output)/(mutant input/wild-type input). Input was determined at four hours post infection. Significance of the results was determined with the Kruskal-Wallis Test (ANOVA) with Dunn's Multiple Comparisons Test and with the Mann-Whitney/Wilcoxon rank sum test. Enumeration of yeast by patch test onto HMM and HMM+hygromycin was also performed and yielded a CI identical to the plating of serial dilutions (data not shown).

Female C57BL/6J mice were purchased from The Jackson Laboratories. Experiments were in accordance with the NIH Guide for the Care and Use of Laboratory Animals and were approved by the Institutional Animal Care and Use Committee at U. C. San Francisco.

## Supporting Information

Table S1 Primers Used in This Study
(0.03 MB DOC)

Figure S1 Iron Regulated Gene Expression of Siderophore Biosynthesis Genes in G186AR. Quantitative RT-PCR of siderophore biosynthesis genes in G186AR (hcLH95). Total RNA from cells grown in the presence or absence of 5 µM FeSO_4_ for 1, 4, and 24 hrs was analyzed for relative transcript levels using QRT-PCR. Transcript levels were normalized to *ACT1* transcript. Relative quantities were normalized to t = 1 hr+Fe values. For the 1, 4, and 24 hr time points, white bars indicate growth in medium supplemented with FeSO_4_ (+Fe), and gray bars indicate growth in the absence of FeSO_4_ (no iron).
(0.33 MB EPS)

## References

[1] Schaible UE, Kaufmann SH (2004). Iron and microbial infection.. Nat Rev Microbiol.

[2] Woods JP (2003). Knocking on the right door and making a comfortable home: Histoplasma capsulatum intracellular pathogenesis.. Curr Opin Microbiol.

[3] Lane TE, Wu-Hsieh BA, Howard DH (1991). Iron limitation and the gamma interferon-mediated antihistoplasma state of murine macrophages.. Infect Immun.

[4] Newman SL, Gootee L, Brunner G, Deepe GS (1994). Chloroquine induces human macrophage killing of Histoplasma capsulatum by limiting the availability of intracellular iron and is therapeutic in a murine model of histoplasmosis.. J Clin Invest.

[5] Cain JA, Deepe GS (1998). Evolution of the primary immune response to Histoplasma capsulatum in murine lung.. Infect Immun.

[6] Howard DH, Rafie R, Tiwari A, Faull KF (2000). Hydroxamate siderophores of Histoplasma capsulatum.. Infect Immun.

[7] Burt WR, Underwood AL, Appleton GL (1981). Hydroxamic acid from Histoplasma capsulatum that displays growth factor activity.. Appl Environ Microbiol.

[8] Timmerman MM, Woods JP (1999). Ferric reduction is a potential iron acquisition mechanism for Histoplasma capsulatum.. Infect Immun.

[9] Nittler MP, Hocking-Murray D, Foo CK, Sil A (2005). Identification of Histoplasma capsulatum transcripts induced in response to reactive nitrogen species.. Mol Biol Cell.

[10] Hwang L, Hocking-Murray D, Bahrami AK, Andersson M, Rine J (2003). Identifying phase-specific genes in the fungal pathogen Histoplasma capsulatum using a genomic shotgun microarray.. Mol Biol Cell.

[11] Haas H (2003). Molecular genetics of fungal siderophore biosynthesis and uptake: the role of siderophores in iron uptake and storage.. Appl Microbiol Biotechnol.

[12] Neilands JB (1995). Siderophores: structure and function of microbial iron transport compounds.. J Biol Chem.

[13] Schrettl M, Bignell E, Kragl C, Sabiha Y, Loss O (2007). Distinct Roles for Intra- and Extracellular Siderophores during Aspergillus fumigatus Infection.. PLoS Pathog.

[14] Oide S, Moeder W, Krasnoff S, Gibson D, Haas H (2006). NPS6, encoding a nonribosomal peptide synthetase involved in siderophore-mediated iron metabolism, is a conserved virulence determinant of plant pathogenic ascomycetes.. Plant Cell.

[15] Haas H, Schoeser M, Lesuisse E, Ernst JF, Parson W (2003). Characterization of the Aspergillus nidulans transporters for the siderophores enterobactin and triacetylfusarinine C.. Biochem J.

[16] Mitsuhashi N, Miki T, Senbongi H, Yokoi N, Yano H (2000). MTABC3, a novel mitochondrial ATP-binding cassette protein involved in iron homeostasis.. J Biol Chem.

[17] Scazzocchio C (2000). The fungal GATA factors.. Curr Opin Microbiol.

[18] Voisard C, Wang J, McEvoy JL, Xu P, Leong SA (1993). urbs1, a gene regulating siderophore biosynthesis in Ustilago maydis, encodes a protein similar to the erythroid transcription factor GATA-1.. Mol Cell Biol.

[19] Haas H, Zadra I, Stoffler G, Angermayr K (1999). The Aspergillus nidulans GATA factor SREA is involved in regulation of siderophore biosynthesis and control of iron uptake.. J Biol Chem.

[20] Zhou LW, Haas H, Marzluf GA (1998). Isolation and characterization of a new gene, sre, which encodes a GATA-type regulatory protein that controls iron transport in Neurospora crassa.. Mol Gen Genet.

[21] Freter R, O'Brien PC, Macsai MS (1981). Role of chemotaxis in the association of motile bacteria with intestinal mucosa: in vivo studies.. Infect Immun.

[22] Taylor RK, Miller VL, Furlong DB, Mekalanos JJ (1986). Identification of a pilus colonization factor that is coordinately regulated with cholera toxin.. Ann Sclavo Collana Monogr.

[23] Howard DH (2004). Iron gathering by zoopathogenic fungi.. FEMS Immunol Med Microbiol.

[24] Lin JS, Wu-Hsieh BA (2004). Functional T cells in primary immune response to histoplasmosis.. Int Immunol.

[25] Schrettl M, Bignell E, Kragl C, Joechl C, Rogers T (2004). Siderophore biosynthesis but not reductive iron assimilation is essential for Aspergillus fumigatus virulence.. J Exp Med.

[26] Mei B, Budde AD, Leong SA (1993). sid1, a gene initiating siderophore biosynthesis in Ustilago maydis: molecular characterization, regulation by iron, and role in phytopathogenicity.. Proc Natl Acad Sci U S A.

[27] Eichhorn H, Lessing F, Winterberg B, Schirawski J, Kamper J (2006). A ferroxidation/permeation iron uptake system is required for virulence in Ustilago maydis.. Plant Cell.

[28] Ramanan N, Wang Y (2000). A high-affinity iron permease essential for Candida albicans virulence.. Science.

[29] An Z, Mei B, Yuan WM, Leong SA (1997). The distal GATA sequences of the sid1 promoter of Ustilago maydis mediate iron repression of siderophore production and interact directly with Urbs1, a GATA family transcription factor.. Embo J.

[30] Yuan WM, Gentil GD, Budde AD, Leong SA (2001). Characterization of the Ustilago maydis sid2 gene, encoding a multidomain peptide synthetase in the ferrichrome biosynthetic gene cluster.. J Bacteriol.

[31] Kragl C, Schrettl M, Abt B, Sarg B, Lindner HH (2007). EstB-mediated hydrolysis of the siderophore triacetylfusarinine C optimizes iron uptake of Aspergillus fumigatus.. Eukaryot Cell.

[32] Bok JW, Keller NP (2004). LaeA, a regulator of secondary metabolism in Aspergillus spp.. Eukaryot Cell.

[33] Bok JW, Noordermeer D, Kale SP, Keller NP (2006). Secondary metabolic gene cluster silencing in Aspergillus nidulans.. Mol Microbiol.

[34] Worsham PL, Goldman WE (1988). Quantitative plating of Histoplasma capsulatum without addition of conditioned medium or siderophores.. J Med Vet Mycol.

[35] Woods JP, Heinecke EL, Goldman WE (1998). Electrotransformation and expression of bacterial genes encoding hygromycin phosphotransferase and beta-galactosidase in the pathogenic fungus Histoplasma capsulatum.. Infect Immun.

[36] Sullivan TD, Rooney PJ, Klein BS (2002). Agrobacterium tumefaciens integrates transfer DNA into single chromosomal sites of dimorphic fungi and yields homokaryotic progeny from multinucleate yeast.. Eukaryot Cell.

[37] Gebhart D, Bahrami AK, Sil A (2006). Identification of a copper-inducible promoter for use in ectopic expression in the fungal pathogen Histoplasma capsulatum.. Eukaryot Cell.

[38] Eisen MB, Spellman PT, Brown PO, Botstein D (1998). Cluster analysis and display of genome-wide expression patterns.. Proc Natl Acad Sci U S A.

[39] Sebghati TS, Engle JT, Goldman WE (2000). Intracellular parasitism by Histoplasma capsulatum: fungal virulence and calcium dependence.. Science.

[40] Schwyn B, Neilands JB (1987). Universal chemical assay for the detection and determination of siderophores.. Anal Biochem.

