# Supplementary material for: Histoplasma Requires SID1, a Member of an Iron-Regulated Siderophore Gene Cluster, for Host Colonization
Source: PLoS Pathog. 2008 Apr 11;4(4):e1000044. doi: 10.1371/journal.ppat.1000044 (PMC2275787; doi:10.1371/journal.ppat.1000044)
Supplement: Table S1 — Primers Used in This Study (0.03 MB DOC) [file ppat.1000044.s001.doc]

# Table S1: Primers Used in This Study

| Gene | **Sense oligo** | **Antisense oligo** |
| --- | --- | --- |
| *SID3* | acacgaacgaaacccatctc | ccatgtggtcctctttagcc |
| *SID4* | gaagatgaggatggcacgat | cgtgtttgggagccttatgt |
| *SID1* | acccttggagcaaattgttg | ctcagaccatggctgctttc |
| *NIT22* | agcacgggctgtgttatctc | tcaacaaggcctttccattt |
| *NPS1* | ctttatacgagggcgagctg | agtggcaagcgtgatttctt |
| *ABC1* | caagccaacagatgcagaaa | atgggtgcctttatcagtgc |
| *OXR* | tgtgaaggttaattcagctctaaac | ggcaccaaaacaccatagc |
| *MFS1* | gctggctacgtcctcaactc | agggaaggatgttgttcca |
| *UBP1* | atgacggattcgacttgacc | gcctgctccatggtaatgtt |
| *ACT1* | gaaggagattaccgctctcg | cgacaacaacgaaaaccttaga |
| *SID5* | gaaggagggggcagaaaa | tcaaagcccattcgtcgt |
| *MFS2* | agctagcgggttggcttt | ctggcctgggtgattgac |
| *SID1* complementation | ggggacaagtttgtacaaaaaagcaggct  ttctcgatactttgctttgacaatttga | ggggaccactttgtacaagaaagctgggtgat  gtataaataaccaaggagaagaacagga |
